# Exercise Intervention on Insomnia in Patients with a Cancer: A Systematic Review of the Literature

**DOI:** 10.3390/cancers16122241

**Published:** 2024-06-17

**Authors:** Chloé Drozd, Elsa Curtit, Valérie Gillet, Quentin Jacquinot, Nathalie Meneveau, Fabienne Mougin

**Affiliations:** 1 Sports Science Faculty, University of Franche-Comté, 25000 Besançon, France; 2 Research Unit SINERGIES, University of Franche-Comté, 25000 Besançon, France; 3 Sleep Medicine Center, Don Du Souffle Association, 25000 Besançon, France; 4 INSERM U1098 Right, University of Franche-Comté, 25000 Besançon, France; 5 Department of Medical Oncology, University Hospital, 25000 Besançon, France; 6 Regional Federative Cancer Institute of Franche-Comté, 25000 Besançon, France

**Keywords:** exercise, supportive care, cancer, insomnia, sleep disturbances, systematic review

## Abstract

**Simple Summary:**

Insomnia is a frequent sleep disorder complaint in cancer patients during and after treatment, and exercise was suggested as a useful non-pharmacological treatment. However, the lack of definitions and the high variability of insomnia measurement tools in the literature do not allow for a clear consensus. This is the first systematic review to evaluate the effects of exercise focused only on insomnia in the cancer population, during and/or after treatment. This systematic review shows significant improvement of better-quality sleep in three of the nine included studies, especially in patients with insomnia at baseline. Less insomnia is associated with aerobic exercise and/or strength training at moderate intensity.

**Abstract:**

Cancer is associated with increased muscle weakness, reduced physical functioning, increased fatigue, but also sleep disturbances, including insomnia, that affect quality of life (QoL). Physical activity demonstrated benefits on functional capacity, resilience and cancer-related fatigue, but there is a paucity of available data regarding its effects on insomnia in patients with cancer. This systematic review aims to examine the efficacy of exercise levels with insomnia in cancer patients. A systematic search was performed for articles published in PubMed and Cochrane Library databases from December 2013 to February 2023. Included studies explored insomnia during or after cancer treatment, with various exercise interventions. The search identified nine studies included in this review. Due to substantial heterogeneity in the interventions across studies, meta-analysis was not performed. Three studies reported positive results for insomnia reduction by self-reported outcomes under a supervised aerobic exercise program alone or combined with strength training. The present systematic review establishes the role of exercise interventions for reducing cancer-related insomnia. Further studies are indeed warranted to improve the level of evidence for exercise interventions for implementation in the care of cancer-related insomnia.

## 1. Introduction

An important but often overlooked side effect of cancer diagnosis and treatment is sleep disturbances, including insomnia, which worsen the QoL of patients [1]. Indeed, the prevalence rate for insomnia in patients with cancer is nearly three times higher than that in the general population [2], and ranges from 30% to 60%, depending on the definition used, the time of assessment, and the measurement tool [3,4]. According to the most recent Diagnostic and Statistical Manual of mental disorders, fifth edition, Text Revision (DSM-5-TR), insomnia is defined as dissatisfaction with sleep quality or quantity characterized by difficulty initiating sleep, sleep maintenance, or early morning awakenings. Insomnia causes significant distress or impairment in daytime functioning and occurs at least three nights per week for at least three months despite adequate opportunity for sleep [5,6]. The etiology of insomnia is complex and multifaceted, particularly in relation to cancer [7]. Spielman’s 3P model outlining the development and maintenance of insomnia posits that pre-disposing, precipitating and perpetuating factors exist, which contribute to the onset and persistence of insomnia [8]. For cancer patients, reduced daytime activity and side effects of treatment, such as increased fatigue, can further compound the risk of developing persistent insomnia [9].

Although hypnotic medications are the most commonly used to treat insomnia, they have some drawbacks, including adverse side effects, risk of dependence and insomnia rebound [2,10]. In addition, in the context of cancer, there is potential reluctance by patients to take additional medications, due to potential interactions with ongoing anticancer medication [11]. Instead, an alternative non-pharmacological treatment approach known as cognitive behavioral therapy for insomnia (CBT-I) has accumulated substantial evidence as an efficacious and durable therapy for insomnia [12,13,14,15,16,17,18]. However, it is not available for many patients, and may be complex to implement in routine clinical practice [19].

Recently, exercise intervention has become an integral part of multidisciplinary supportive care, and it is now seen as a new paradigm to improve patient survival and QoL [20,21,22]. Nevertheless, insomnia is still poorly investigated and there is a paucity of research about the relationship between exercise and sleep in cancer patients. Yet, in the general population, exercise has a beneficial impact on insomnia [23]. In the context of cancer, no consensus exists to determine the extent to which exercise can be accredited as a sleep-enhancing intervention in cancer patients. 

In this paper, we propose an overview of the state-of-the-art physical exercise interventions and insomnia in the management of cancer patients. We review available evidence for selecting exercise as a non-pharmacology therapy for cancer patients with insomnia. 

## 2. Methods

### 2.1. Eligibility Criteria

Studies were eligible if they were prospective randomized or non-randomized, published in English or French and evaluated insomnia outcomes in pre- and post-exercise intervention. The inclusion criteria were studies involving patients (18 years and older), with any type of cancer diagnosis and at any stage of cancer care (i.e., undergoing or post-treatment).

Exercise interventions were considered eligible if they included the following criteria: aerobic exercise, strength training or a combination of both. The intervention could be supervised and/or home-based, whatever the Frequency, Intensity, Time, Type, Duration (FITT-D) of exercises. Studies were not eligible if mind-body activities (i.e., yoga, Tai-Chi, Qigong) were proposed, due to highly heterogeneous methodologies. 

To be included, trials had to evaluate insomnia as primary or secondary outcome using self-reported or objective assessments. 

### 2.2. Information Sources and Search

Eligible studies were identified by a systematic search in PubMed and Cochrane Library databases from December 2013 to February 2023 (Figures S1 and S2). Participants’ characteristics, study design, exercise interventions and results were selected following the PICO (population, intervention, comparator, and outcomes) as follows: (1) P (population), neoplasm*, cancer*, malignant*; (2) I (intervention), exercise, physical activity*, aerobics, gymnastics, training, walking, dancing, dance, running, jogging, sport, swimming, cycling, physical education, rehabilitation; (3) C (comparator), all other interventions; and (4) O (outcomes), insomnia. 

The search was conducted using a combination of Medical Subject Headings (MeSH) and was adjusted to the characteristics of each database. Each search term was linked with “OR”, “AND” to search for relevant literature. The Preferred Reporting Items for Systematic Reviews and Meta-Analyses (PRISMA) protocol was applied [24].

### 2.3. Data Collection Process

Once the search was done, and after duplicates were removed, the titles and abstracts of identified studies were examined independently by three researchers to identify studies meeting the selection criteria. Then, the full text of selected studies was assessed by three investigators. Among identified papers that originated from the same program or dataset, all reports were included. The methodological quality of Randomized Controlled Trials (RCTs) was examined using the risk of bias criteria recommended by the Cochrane Collaboration using Risk of Bias 1.0 tool (RoB1) [25]. Three independent reviewers also carried out the scoring. 

## 3. Results

### 3.1. Study Selection

**Figure 1:**
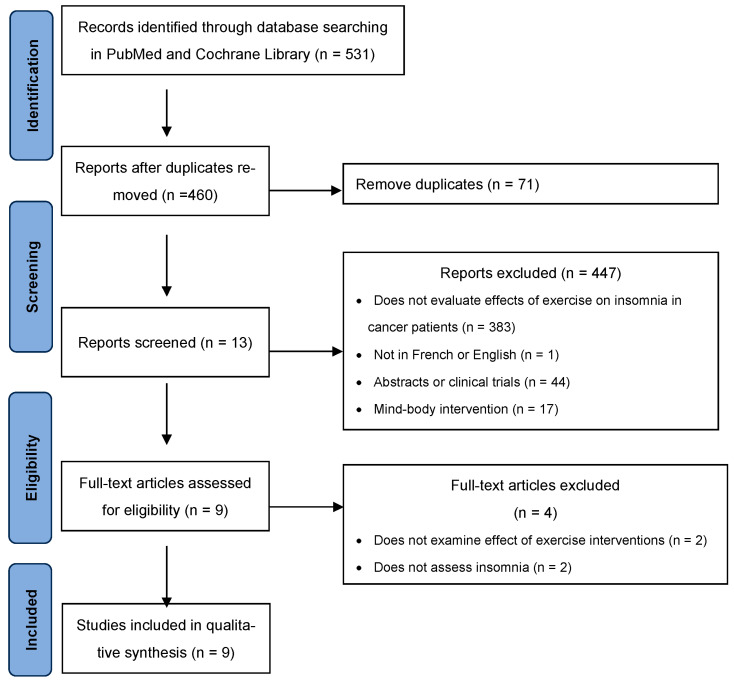
PRISMA flow diagram.

A total of 531 records, published in PubMed (*n* = 327) and Cochrane Library (*n* = 204) were screened. After removal of 71 duplicates, 460 were initially screened. Of these 460 papers, 447 were excluded based on our exclusion criteria (Figure 1). The full text of the remaining thirteen articles was retrieved and reviewed, but four of these were subsequently excluded because either the effects of exercise intervention or insomnia were not assessed. A final total of nine studies were included in the review.

### 3.2. Study Characteristics

Table 1 summarizes the characteristics of the nine studies included. Among these, four were RCTs [26,27,28,29], two were non-RCTs [30,31], one was a cohort study [32], and two were case series studies [33,34].

Four studies used a 3-arm design, including one control group [healthy control group [32] or usual care [26,28,29]. In three of them, the interventional groups performed various forms of exercise [26,28,29], but in the study by Colledge et al. [32], the exercise intervention was identical for the three different samples (aneurysmal subarachnoid haemorrhage, meningioma patients and control group). Two studies described a 2-arm design, with one physical training group, compared to either a group participating in CBT-I or a group receiving health education [30]. Two studies used a single-arm design that offered only an exercise intervention [33,34]. Yamada et al. [31] provided aerobic and resistance training for paired versus individually trained cancer patients. 

Across all studies, the sample sizes ranged from 16 to 75 patients: two studies included 72 and 75 patients [29,33], two included 16 and 18 participants [28,34], and five studies included 28 to 48 patients [26,27,30,31,32]. 

### 3.3. Quality Evaluation

**Figure 2:**
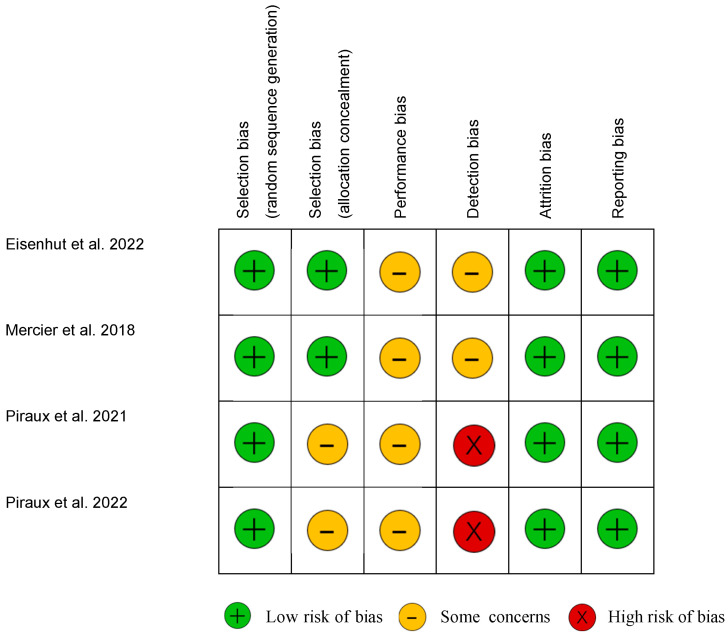
Risk of bias in randomized controlled studies [26,27,28,29].

The risk of bias for RCTs is summarized in Figure 2. All studies had a low risk of bias for random sequence generation, attrition and selective outcome reporting. Two studies had insufficient information for allocation concealment. The four RCTs did not have sufficient information to determine the risk of bias for performance and detection, excepted a high risk in studies by Piraux et al. [28,29] about the blinding of the outcome assessment. 

### 3.4. Participants 

The mean age across studies varied from 51 to 59 years old, with a standard deviation between 10 and 12.5 years for most studies. Seven studies (78%) included both men and women [26,27,28,30,32,33,34], four of which (57%) had mostly women. One study was exclusively conducted among women [31], and one did not specify the gender [29]. 

The populations of five studies (56%) included mixed cancer sites, of which breast cancer was the most frequent (ranging from 53% to 92.9%) [27,30,31,33,34], except for Charles et al. [34], in which 68.75% of participants had melanoma. Four studies (44%) were conducted with a single cancer site (meningioma, glioma, prostate or rectal cancer). Four studies included patients in post-adjuvant treatment (except ongoing hormone therapy) with a mean time from last treatment varying from 20.5 days to 10 years. Four other studies investigated only patients undergoing cancer treatment at the time of the study (i.e., immunotherapy, radiation therapy, adjuvant or neoadjuvant chemotherapy), and one study (11%) included patients undergoing treatment and those post-treatment (within 6 months of treatment completion). 

Cancer stage was not systematically reported, but three studies included stages 0 to IV [26,27,28]. In the studies by Colledge et al. [32] and Mercier et al. [27], patients were taking medications such as antidepressants (13%), hypnotic or anxiolytic medications (45 and 47.5% respectively), and Sheehan et al. [30] reported that 56.7% of participants were taking anti-inflammatory drugs. Finally, data were available about treatments related to sleep in only two studies (33%).

Two studies focused on fatigue complaint as an inclusion criterion, with patients presenting a level ≥4 on a 10-point visual analogue scale [34], or a score <45 points on the Functional Assessment of Cancer Therapy-Fatigue (FACT-F) [30]. In Mercier et al.’s study [27], participants were considered eligible if they had insomnia symptoms with a score ≥8 on the Insomnia Index Severity (ISI) questionnaire (Table 1).

### 3.5. Exercise Interventions

Table 2 provides a summary of exercise program interventions and the main results obtained for each study. Exercise interventions varied widely across studies. Six trials (67%) offered supervised interventions [26,28,29,31,33,34], of which one was delivered by videoconference [34]. Sheehan et al. [30] tested supervised sessions twice weekly for the first 5 weeks of the program, then once weekly for the remaining 5 weeks, during which home-based exercise sessions were increased. In the study by Colledge et al. [32], patients had a 12-week exercise program, with three to five sessions per week, only one of which was supervised. The study by Mercier et al. [27] focused only on home-based exercise. 

Only one study tested aerobic exercise training [27] and four (44%) assessed a combination of aerobic and resistance training [26,31,33,34], to which relaxation, stretching, balance and flexibility exercises were added [31,34]. Piraux et al. [28,29] published two trials that used resistance training compared to high intensity interval training (HIIT). Two studies tested aerobic training associated with flexibility, motor skills learning tasks, taught behavioral skills [32] or stretching [30]. For aerobic exercise interventions, a cycle ergometer, treadmill, or a combination of different aerobic exercises (e.g., walking, jogging, cycling or swimming) was used. Studies including resistance training used body weight, machines, resistance bands or a combination of different modalities. HIIT sessions were delivered on cycle ergometer or a cross trainer.

The method applied to define the intensity of exercise differed from one trial to another. The percentage of maximum (from 55 to 85% or more) or reserve (from 40 to 60%) heart rate was used in five studies (56%) [28,29,30,31,32]. Rating of Perceived Exertion (RPE, 6–20) with the Borg scale [26,30] or modified (0-10) Borg scale [27,28,29,31] was applied in six studies, while the percentage of 1-Repetition Maximum (RM) (40 to 60% of 1-RM) was used in the study by Yamada et al. [31]. One study [33] did not specify exercise intensity and another [34] indicated a “moderate” intensity according to the Guidelines of the National Comprehensive Cancer Network and American College of Sports Medicine.

Exercise frequency ranged from two to five sessions per week, except for Charles et al. [34], where participants were instructed to follow the French national recommendations of at least 150 min of moderate-intensity exercise per week. Sessions lasted between 20 and 150 min. Regarding the duration of the exercise program, most included studies had interventions lasting from 5 to 12 weeks, except for one, for which the intervention lasted 6 months [34]. In most studies, no follow-up measure [26,28,29,31,33] was performed, while four studies performed follow-up measures at 3 to 6 months after the end of intervention [27,30,32,34].

### 3.6. Adherence and Compliance 

Only three studies reported attendance at exercise sessions, which varied from 78% to 93.5% [28,29,34]. Most studies reported an adherence rate (percentage of participants who completed study measures) between 87% and 100%, while two studies reported attendance <71% [32,33]. During follow-up, adherence rates were between 56.2% and 89.4% [27,30,32,34] (Table 2).

### 3.7. Sleep Outcomes

Insomnia was the primary outcome in two studies [27,33], and was reported as a secondary outcome in the seven other studies [26,28,29,30,31,32,34]. Insomnia was predominantly measured by self-report scales. The Athens Insomnia Instrument (AIS) was proposed in only one study [33]. Soldatos et al. [35] suggested a cutoff score of 6, which correctly distinguished between insomnia patients and controls in 90% of cases. The Insomnia Severity Index (ISI) is an instrument to assess insomnia severity and has been validated in patients with cancer [36]. It was used in eight studies (89%). Its total score ranges from 0 to 28, with higher values indicating more severe insomnia. A reduction of 6 points is representative of a clinically meaningful improvement in individuals with primary insomnia [37].

Two studies used electroencephalography (EEG) and actigraphy as objective measures of insomnia. In the study by Mercier et al. [27], participants wore an actigraphic device for seven consecutive 24-h periods. One objective nighttime EEG was recorded in the study by Colledge et al. [32] (Table 2).

### 3.8. Effect of Exercise Interventions on Insomnia Outcome at Baseline

Among the nine studies included, eight used the ISI questionnaire. The mean score for five of them varied from 6.89 to 16 points. Accordingly, three studies reported a patient group with sub-clinical insomnia (8.19 to 11.5 points) [26,31,32], and in two studies, patients had clinical insomnia (15.2 to 16 points) [27,30]. In the study by Eisenhut et al. [26], only patients in the strength training group did not have insomnia, although their score was at the clinical margin (6.89 points). Piraux et al. [28,29] described only median ISI scores, which ranged from 5.5 to 8.5. In the study by Kozik et al. [33], patients had a mean score (evaluated by AIS) of 9.5 at baseline, indicating the presence of insomnia. In Charles et al. [34], six patients (42.9%) did not have insomnia, four (28.6%) had subthreshold insomnia and four (28.6%) had clinical insomnia (Table 2). 

### 3.9. Pre- and Post-Intervention Insomnia Outcome

Kozik et al. [33] showed a significant decrease of insomnia after 10 weeks of aerobic and strength training intervention. Charles et al. [34] described only exploratory statistical analyses, precluding conclusions about the presence or absence of insomnia. 

In the study by Eisenhut et al. [26], insomnia scores were reduced over time in the endurance and active control conditions with large and medium effect sizes respectively, but they increased in the strength condition after 6 weeks of exercise intervention.

In the study by Mercier et al. [27], aerobic exercise trained patients and the CBT-I group improved their insomnia post-intervention with a moderate effect size, switching from “clinical” to “sub-clinical insomnia”. Results of the non-inferiority analysis showed that the exercise intervention was significantly inferior to CBT-I in reducing insomnia symptoms post-treatment, as measured with the ISI. However, results at 3- and 6-month follow-up indicated that the exercise intervention was not significantly inferior to CBT-I in reducing ISI scores.

The results reported by Piraux et al. [28,29] showed no significant time or group effect between resistance, HIIT exercise intervention and control groups. 

In the study by Sheehan et al. [30], insomnia decreased in the exercise group, with a significant time and group effect, after 26 weeks of follow-up compared to the health education group.

Colledge et al. reported only descriptive statistics. Insomnia scores decreased among all groups but were lower in the control group across all measurement points [32]. Yamada et al. showed no time effect pre- vs. post-intervention in both groups [31]. Nevertheless, in paired trained patients, a significant decrease in insomnia (3.6 points) was observed at mid-intervention. 

Finally, Kozik et al., Mercier et al. and Sheehan et al. found a positive effect of an exercise program on insomnia evaluated by ISI in post-intervention [27,30,33]. 

In a study by Colledge et al., the exercise program did not have notable effects on the objective sleep parameters as assessed by EEG in meningioma patients. In the descriptive results, the very short Sleep Onset Latency (SOL) at baseline in the meningioma group, increased in post-test and during follow-up but remained shorter than in the other two groups. Sleep parameters such as Total Sleep Time (TST), Wake After Sleep Onset (WASO), light sleep, slow wave sleep (SWS) and Rapid Eye Movement (REM) sleep did not change after intervention or at 6-months follow-up [32].

Mercier et al. showed only significant time effects in morning awakenings (reduction of approximately 5 min) and WASO (approximately 7 min) from pre- to post-treatment in the CBT-I group. Sleep efficiency was below the clinical threshold of 85%, at each time point [27]. 

## 4. Discussion

This literature review summarizes the available empirical evidence from four RCTs and five non-RCTs containing data from 2013 until 2023. Considering the recent attention given to the impact of exercise interventions on sleep in cancer patients, the studies selected for this review specifically target insomnia as either a primary or secondary objective.

The relationship between exercise and insomnia is poorly assessed in the context of cancer. Among the nine selected studies, only three found that a supervised and/or home-based exercise intervention significantly reduced insomnia, as assessed by the ISI, and two of them compared a trained exercise group to a control group [30] or a CBT-I group [27]. Benefits of exercise were also found in the single-arm study of Kozik et al. [33]. These studies used either aerobic exercise only [27] or aerobic exercise combined with strength training [30,33]. The exercise program varied in duration from 6 to 10 weeks, and from 20 to 150 min per session, with a frequency of two [30,33], or three to five sessions per week [27]. In the study by Kozik et al. [33], the intensity was not described, whereas Mercier et al. and Sheehan et al. used both the Rating of Perceived Exertion [score varying from 3 to 5 [27] and 11 to 13 [30]], and a percentage of maximal heart rate (66 to 85%) for one of them [30]. However, considering the other results did not have positive effects on insomnia, this could be explained by heterogeneity in terms of participants or exercise interventions between or within included RCTs. Indeed, most interventions were supervised [26,28,29,31,34], except in the study by Colledge et al. [32], and duration of programs varied between 5 and 12 weeks [26,28,29,31,32], with one lasting 6 months [34]. The intensity and frequency of sessions also varied from one study to another, while only three described attendances at the exercise session. In the literature, there is a small body of evidence showing that some clinical (e.g., baseline severity of sleep disturbances), and personal (e.g., body mass index) and cancer-related (e.g., chemotherapy, cancer type)) factors alleviated the effects of exercise interventions on health-related QoL, and by extension, on insomnia [38]. Furthermore, the characteristics of the exercise intervention (e.g., dose, type, duration) have also been found to influence the effects of the intervention on sleep [39]. However, the moderating analyses were generally not planned a priori and were often conducted with insufficient statistical power. 

Overall, positive insomnia outcomes are associated with aerobic exercise and/or strength training, with a moderate intensity. Thus, it is difficult to have a clear consensus about dose of exercise required to improve cancer-related insomnia.

Some other points are noteworthy. The women’s history of sleep deficiency, or present sleep habits, were not evaluated in any study. Therefore, it is possible that studies without significant results could be partially explained by a celling effect. In the study by Piraux et al. [29], patients did not have insomnia, or were at the clinical margin at baseline. In the study by Mercier et al. [27], patients were only included if they had a score of 8 or higher on the ISI. In the study of Sheehan et al. [30], patients needed to experience ongoing fatigue with a score <45 points on the Functional Assessment of Cancer Therapy-Fatigue. However, insomnia and cancer-related fatigue are common symptoms in oncology and a relationship between these two comorbid symptoms has been reported [40,41,42]. 

Mercier et al. and Sheehan et al. reported that all participants had “clinical insomnia” with the same ISI scores between exercise and control [30] or exercise and CBT-I groups [27]. In Kozik et al. [33], all patients had insomnia, as evaluated by the AIS, a self-report psychometric instrument corresponding to criteria for the diagnosis of insomnia based on the International Classification of Diseases 10th Revision (ICD-10), but which does not address the chronicity and frequency of insomnia. In those three studies, all patients had insomnia before intervention, and their scores were reduced by a mean of 6 points post-intervention, what represents a clinically meaningful difference [37]. Accordingly, an exercise intervention appears to provide greater effects when insomnia is severe. 

Only three studies reported use of hypnotics or other treatments (i.e., anxiolytic or antidepressant) that could partially explain the reduction in insomnia [27,30,32]. 

Although polysomnography or actimetry are not routinely required for insomnia diagnosis, they can be considered as part of a detailed exploration of sleep, according to the patient’s clinical condition [43]. However, the two studies that evaluated objective sleep using EEG [32] or actimetry [27] did not report an improvement in sleep parameters (TST, WASO, light sleep, SWS, REM sleep) after the exercise intervention. It is reported that satisfaction with sleep, rather than objectively assessed sleep quality itself, appears to be more important for well-being [44]. From this perspective, it can be cautiously recommended that exercise remains a beneficial intervention for cancer patients complaining about poor sleep, regardless of the absence of significant results or large effect sizes on objective sleep parameters. We would like to point out that objectively assessed sleep has been sparsely studied in this population, so the understanding of how this domain may evolve over time is limited.

The review by Takemura et al. [45] was the first to adopt stringent inclusion criteria and only included studies with participants above significant thresholds for sleep disorders. The main results suggest that aerobic exercise interventions significantly improved sleep for cancer patients with an effect that remained significant after 3 to 6 months. 

Despite high variability in exercise type, dose, and study methods, Matthews et al. [46] showed an improvement in sleep deficiency in 73% of selected studies and all walking interventions resulted in positive sleep outcomes. For Kreutz et al. [47], exercise (aerobic and/or resistance) improved sleep quality, especially the PSQI subscales for “sleep quality” and “sleep disturbances” in breast cancer patients. Overall, objective sleep parameter assessments by actigraphy represented a minority of outcomes, with discordant results, and PSQI was often the most used tool to evaluate sleep quality [45,46,47,48,49]. A recent meta-analysis by Gururaj et al. [50] showed significant improvement for PSQI outcome and WASO with exercise. Moreover, when both objective and subjective sleep measures were evaluated, significant improvements in the subjective sleep perception were reported without significant effects on actigraphy parameters [48,49]. 

## 5. Study Strengths and Limitations

This review is the first to investigate the effects of exercise interventions on insomnia in cancer patients. Studies were selected only focusing on insomnia. 

However, this review has some limitations that deserve to be underlined, in particular, the small number of included studies, with only three that reported positive results on cancer-related insomnia. In the RCTs and non-RCTS analyzed over the last 10 years, individualization and adherence to the exercise program are difficult to assess, due to the heterogeneity of cancers. Finally, very few studies reported objective sleep measurements.

## 6. Conclusions

Insomnia is associated with cancer-related comorbid conditions such as emotional distress, anxiety/depressive symptoms and low QoL. While the relationship between exercise and insomnia in the context of cancer remains understudied, our analysis reveals promising outcomes in select studies. Notably, aerobic exercise and/or strength training, often administered over a duration of 6 to 10 weeks, have shown positive effects on reducing insomnia severity in three studies. This review underscores the potential of exercise interventions as a valuable adjunctive therapy for addressing insomnia in cancer patients.

The burden of insomnia in patients with cancer remains to be addressed and requires a call to action for the evaluation of the potentially modifiable factors that might contribute to reducing cancer morbidity. 

## 7. Future Directions

Although the optimal dosage of exercise necessary to have a beneficial effect on insomnia in patients with cancer warrants further investigation, the present analysis allows better targeting of the mechanisms linking exercise with insomnia in these patients. There is nevertheless a need to systematically include follow-up assessments to determine whether or not the beneficial effect of exercise on insomnia symptoms is sustainable in the long-term. 

Our currently ongoing trial, whose main objective is to assess the effects of an exercise program combining high and moderate intensities on insomnia outcome in non-metastatic breast cancer patients during chemotherapy and after a follow-up of 3 months post-exercise intervention, should provide more information regarding the optimal exercise strategy to reduce clinical levels of insomnia symptoms in patients undergoing treatment [51].

## Figures and Tables

**Table 1:** Studies characteristics.

First Author et al. Year	Sample *N* = Total Sample M = Mean Age Exercise Intervention, *n* = xx UC/Other Group, *n* = xx	Study Design	Gender (m:f)	Cancer Site (%)	Cancer Stage (%)	Treatment Status	Line of Treatment Mean Time Since Treatment	Use of Hypnotic/Anxiolytic Medication
Charles et al. 2021 [34]	*N* = 16 M = 54 ± 12.2 EX group: 16	Monocentric single-arm feasibility trial	8:8	Melanoma (68.75%) Lung (18.75%) Other (12.5%)	NR (more than two-thirds treated for metastatic melanoma)	To be starting or undergoing immunotherapy - Nivolumab: 9 (56.25%) - Pembrolizumab: 7 (43.75%)	Line of treatment: - First: 11 (68.75%) patients - Second and third: 3 (18.75%) patients - Third: 2 (12.5%) patients Mean time since the beginning of therapy: 8 ± 8.2 years	NR
Colledge et al. 2018 [32]	*N* = 48 M = 58.5 ± 12.4 aSAH group, *n* = 15 Meningioma group, *n* = 16 Healthy control group = 17	Exploratory intervention study	18:30	Meningioma	NR	- aSAH patient: after surgical or endovascular intervention - Meningioma patient: after surgical resection No patient from either group was undergoing RX	Line of treatment: NR Mean time to entry in the study after surgery: - aSAH: 44 ± 28.9 months - Meningioma: 21 ± 16.4 months	Use of antidepressants: - aSAH group: 2 (13%) patients - Meningioma group: 1 (6%) patient - Healthy control group: 1 (6%) patient
Eisenhut et al. 2022 [26]	*N* = 29 M = 52.1 ± 12.5 Endurance training group, *n* = 10 Strength training group, *n* = 11 Active control group (UC + sharing experiences from their daily lives), *n* = 8	RCT	44.8%:55.2%	High grade glioma	III (24.1%), IV (75.9%)	After neurosurgical tumor resection or biopsy and undergoing RX and/or CX. - Endurance training group: 10 patients with combined therapy - Strength training group: 10 with combined therapy vs. 1 with RX only - Active control group: 7 with combined therapy vs. 1 with RX only	NR	NR
Kozik et al. 2018 [33]	*N* = 75 M = 59 ± 10 EX group, *n* = 75	Single-arm observational study	13:62	Breast (57.3%), lung (16%), other (26.7%)	NR	Ongoing chemoradiotherapy or within 6 months from completion	NR	NR
Mercier et al. 2018 [27]	*N* = 41 M = 57.1 EX group, *n* = 20 CBT-I group, *n* = 21	RCT	9:32	Breast (53.7%), Gynecologic (7.3%), Lymphoma (7.3%), Prostate (14.6%), Head and neck (9.8%), Other (7.3%)	0 (4.9%), I (39%), II (26.8%), III (14.6%) Unknown (14.6%)	Post adjuvant treatment (except HX) - EX group: 80% surgery, 30% CX, 100% RX, 15% brachytherapy, 30% current HX - CBT-I: 76.2% surgery, 42.9% CX, 100% RX, 4.8% brachytherapy, 42.9% current HX	Line of treatment: NR Mean time (from last RX at baseline): - EX group: 20.5 ± 24.5 days - CBT-I: 30.4 ± 55 days	- CBT-I group: 10 (47.6%) used hypnotic and anxiolytic medication at baseline with an average frequency of use of 3.0 (3.0) nights per week - In Ex group: 9 (45%) used hypnotic and anxiolytic medication at baseline with an average frequency of use of 2.1 (3.0) nights per week
Piraux et al. 2021 [29]	*N* = 72 M = None RES training group, *n* = 6 HIIT group, *n* = 6 UC, *n* = 6	RCT	NR	Prostate	NR	During RX (at least 25 scheduled radiation sessions with or without HX) - RES training group: 7 (29.2%) previous prostatectomy, 20 (83.3%) HX - HIIT group: 4 (16.7%) previous prostatectomy, 21 (87.5%) HX - UC: 3 (12.5%) previous prostatectomy, 17 (70.8%) HX	Line of treatment: NR Days between the start of HX and the start of RX: - RES training group: 98 - HIIT group: 104 - UC: 116	NR
Piraux et al. 2022 [28]	*N* = 18 M = None RES training group, *n* = 6 HIIT group, *n* = 6 UC, *n* = 6	RCT	13:5	Rectal	II (33.3%) III (66.7%)	During neoadjuvant chemoradiotherapy followed by surgery A total dose of 45.0 Gy in 25 fractions over 5 weeks with concurrent oral capecitabine (dose of 1500 mg/m^2^ twice daily on days of RX) or continuous intravenous infusions of 5-fluorouracile (dose of 225 mg/m^2^ daily, five days per week)	NR	NR
Sheehan et al. 2020 [30]	*N* = 37 M = 55 ± 2 EX group, *n* = 19 Health education group, *n* = 18	Non-RCT	4:33	Breast (81%), Prostate (11%), lung (6%) Endometrial (5%), Esophageal (5%), Multiple myeloma (6%), Cervical (6%)	NR	After surgery and CX-RX treatment, ongoing HX - EX group: 86% surgery, 79% RX, 86% CX, 80% HX - Health education group: 94% surgery, 89% RX, 68% CX, 67% HX	Line of treatment: NR Mean time: - EX group: 2.9 ± 0.5 years - health education: 1.6 ± 0.4 years	56.7% of patients used anti-inflammatory
Yamada et al. 2021 [31]	*N* = 28 M = 58 ± 11 EX in pairs, *n* = 14 EX individually, *n* = 14	Experiential study	0:28	Breast (92.9%), Ovarian (3.6%), Lymphoma (3.6%)	NR	To have completed clinical cancer treatments at least 3 months previously	NR	

aSAH: aneurysmal subarachnoid haemorrhage; CBT-I: cognitive behavioral therapy for insomnia; CX: chemotherapy; EX: exercise; HIIT: high intensity interval training; HX: hormonal therapy; NR: not reported; RCT: randomized controlled trial; RES: resistance training; RX: radiotherapy; UC: usual care.

**Table 2:** Summary of exercise program interventions and main results.

Studies	Aim * Criteria about Sleep/Fatigue	Intervention	Components and Intensity	Assessment Time	Adherence	Insomnia Outcome	Effect on Sleep
Charles et al. 2021 [34]	To evaluate the feasibility and the acceptability of a videoconference-based 6-month program promoting physical activity * To report a level of fatigue ≥ 4 on a 10-point visual analogous scale	EX group	Type: [Supervised by videoconference] articular mobilization, aerobic and resistance exercises, relaxation or stretching Frequency: 150 min/week Intensity: moderate Time: 45 to 60 min Duration: 6 months	Before (T1), 6 months at the end of the program (T2), and 3 months later (T3)	Adherence rate: 87.5% (at T2) Avg number of supervised sessions: 20.8 ± 4.8; 87% of planned sessions	ISI	**Secondary outcome** Descriptive statistics: - T1: 6 (42.9%) patients did not have insomnia, 4 (28.6%) had subthreshold insomnia and 4 (28.6%) had clinical insomnia - T2: 7 (53.8%) patients did not have insomnia, 3 (23.1%) had subthreshold insomnia and 3 (23.1%) had clinical insomnia - T3: 5 (55.6%) patients did not have insomnia and 4 (44.4) had clinical insomnia
Colledge et al. 2018 [32]	To compare the effects of an exercise program in aSAH population with another patient group, and a group of healthy controls	3 EX groups	Type: [supervised once a week and unsupervised for the others] walking techniques, flexibility and motor skill learning tasks, and taught behavioral skills Frequency: 3–5 times/week Intensity: 55–65% of max HR (first 4 weeks), 65–75% (weeks 5–8), 75–85% (last 4 weeks) Time: 30 to 45 min Duration: 12 weeks	Baseline (1 week before intervention), after intervention (12 weeks), and 6-month follow-up	Adherence rate: 72% (at 12 weeks) and 67% (at 6 months follow-up)	ISI EEG	**Secondary outcome** Descriptive ISI scores decreased among all groups at pre-post-test, 6 months follow-up - aSAH group: 8.33 ± 5.11 at baseline to 8.13 ± 5.41 in post intervention to 6.93 ± 4.18 at follow-up - Meningioma group: 8.19 ± 4.34 at baseline to 7.38 ± 4.77 in post intervention to 7.13 ± 4.35 at follow-up - Healthy control group: 3.76 ± 2.44 at baseline to 4.09 ± 2.87 in post intervention to 4.06 ± 2.44 at follow-up Large ES for Time x Group founded for insomnia (ISI) Descriptively meningioma group had shorter SOL than other groups across every time point - aSAH group: 23:59 ± 15:20 at baseline to 19:08 ± 11:17 in post intervention to 20:53 ± 10:42 at follow-up - Meningioma group: 06:23 ± 05:38 at baseline to 10:53 ± 11:09 in post intervention to 13:05 ± 11:15 at follow-up - Healthy control group: 17:52 ± 15:43 at baseline to 16:40 ± 16:04 in post intervention to 17:43 ± 16:18 at follow-up Large ES founded for Group for the variable SOL (baseline to follow-up)
Eisenhut et al. 2022 [26]	To investigate the impact of endurance and strength training on symptoms of depression, feelings of stress and anxiety, fatigue, insomnia and physical fitness, compared to an active control condition	Endurance training group Strength training group	Type: [Supervised] on treadmill or bicycle Frequency: twice weekly Intensity: RPE at 11-14 Time: 35 to 45 min Duration: 6 weeks Type: [Supervised] weightlifting and resistance exercises, 3–5 sets of 10–15 rep Frequency: twice weekly Intensity: RPE at 11-15 Time: 35 to 45 min Duration: 6 weeks	Baseline, week 3 (mid-program), and week 6 (end of program)	Adherence rate: 93.16% (at 6 weeks)	ISI	**Secondary outcome** Descriptive statistics: - Endurance training group: ISI scores decreased: 11.50 ± 2.73 at baseline to 6.50 ± 3.35 at week 3 to 7.00 ± 2.71 at week 6 *Large ES (baseline* vs. *week 6)* - Strength training group: ISI scores increased: 6.89 ± 6.02 at baseline to 10.56 ± 6.87 at week 3 to 9.56 ± 7.94 at week 6 *Small ES (baseline* vs. *week 6)* - Active control group: ISI scores decreased: 9.25 ± 2.90 at baseline to 7.75 ± 2.03 at week 3 to 7.75 ± 2.03 at week 6 *Medium ES (baseline* vs. *week 6)*
Kozik et al. 2018 [33]	To determine if a structured, supervised outpatient exercise program specifically for cancer patients would be associated with improvements in insomnia and depression after attending for 10-weeks	EX group	Type: [Supervised] cardiovascular circuit training, strength training Frequency: twice weekly Intensity: None Time: 90 min Duration: 10 weeks	Baseline, after intervention (week 10)	Drop-out rate: 46.7%	Athens Insomnia Instrument	**Primary outcome** Significant decrease of ISI scores: 9.5 ± 3.7 at baseline to 6.3 ± 3.5, *p* < 0.01
Mercier et al. 2018 [27]	To assess the efficacy of a 6-week home-based aerobic exercise program compared to that of a 6-week self-administered cognitive-behavioral therapy for insomnia (CBT-I) to improve sleep in cancer patients * To have insomnia symptoms, as indicated by a score of 8 or greater on the ISI	EX group	Type: [Home-based] individualized aerobic training: brisk walking, jogging, swimming or a combination of different aerobic EXs Frequency: 3–5 times/week Intensity: RPE at 3 to 5 Time: 20 to 30 min until 150 min Duration: 6 weeks	Baseline (pre-treatment), week 6 (post treatment), 3- and 6-months follow-ups	Drop-out rate: 7.3%	ISI Actigraphy	**Primary outcome** - EX group: ISI scores decreased at each time to 16.0 (1.3) at pretreatment to 12.1 (1.7) in posttreatment to 10.4 (1.7) at 3-months follow-up to 9.4 (1.3) at 6-months follow-up *Significant time effect, p < 0.01, moderate ES (pretreatment to 6-month follow-up)* - CBT-I group: ISI scores decreased to 14.8 (1.1) at pretreatment to 10.3 (1.3) in posttreatment, increased to 12.6 (1.2) at 3-months follow-up and decreased to 11.8 (1.2) at 6-months follow-up *Significant time effect, p = 0.04, moderate ES (pretreatment to 6-month follow-up)* NS group x time interaction founded but marginally (*p* = 0.06) EX intervention was significantly inferior to CBT-I at post-intervention but was non-inferior at follow-up No significant effects suggesting that both interventions had a modest impact on participants’ objective sleep. Only significant time effects from pretreatment to posttreatment were obtained in the CBT-I group only on early morning awakenings (reduction 5 min; *p* = 0.01) and WASO (reduction 7 min; *p* < 0.01).
Piraux et al. 2021 [29]	To investigate the effects of HIIT and RES training compared to UC on CTRF, QoL, depression, daytime sleepiness, insomnia, sleep quality, functional exercise capacity and executive function in prostate cancer patients during radiotherapy.	RES group HIIT group	Type: [supervised] Resistance training (i.e., 8 exercises of body wait, resistance bands, dumbbells, 1-3 sets of 8–12 rep) Frequency: 3 times/week Intensity: RPE at 4–6 Time: 30 to 40 min Duration: 5 or 8 weeks Type: [Supervised] on cycle ergometer (60-s work interval at 90–100 rev/min at ≥85% of THRmax and 60-s active rest at 50–60 rev/min) Frequency: 3 times/week Time: 26 to 40 min Duration: 5 or 8 weeks	Baseline (10 days before RX) and after last fraction of RX	Drop-out rate: 7.69% Attendance at EX sessions: 93.5% in HIIT group and 91.4% in RES group	ISI	**Secondary outcome** Descriptive statistics: - UC group: the median [Q1–Q3] scores were the same at baseline 8.0 [4.5; 10.8] and after intervention 8.0 [2.0; 11.8], with mean change (95% CI) from T0 to T1 at -0.7 (−2.6; 1.3) - HIIT Group: the median scores were 7.5 [4.3; 10.0] at baseline and decreased at 6.0 [2.3; 10.8] after intervention, with mean change from T0 to T1 at −0.5 (−2.1; 1.2) - RES group: the median scores were 5.5 [2.8; 8.5] at baseline and increased at 6.0 [3.3; 9.5] after intervention, with mean change from T0 to T1 at 0.5 (−1.7; 2.7) *NS difference between 3 groups after exercise program for ISI scores*
Piraux et al. 2022 [28]	To determine the feasibility of HIIT and RES during NACRT in rectal cancer patients.	RES group HIIT group	Type: [Supervised] resistance training (i.e., 8 exercises of body wait, resistance bands, dumbbells, 1–3 sets of 8–12 rep Frequency: 3 times/week Intensity: RPE at 4–6 Time: 30 to 40 min Duration: 5 weeks Type: [Supervised] on cycle ergometer or cross-trainer (60-s work interval at 90–100 rev/min at ≥85% of THRmax and 60-s active rest at 50–60 rev/min) Frequency: 3 times/week Time: 26 to 40 min Duration: 5 or 8 weeks	Baseline (10 days before NACRT) and after last fraction of RX	Adherence rate: 100% Attendance at EX sessions: 92% in HIIT group and 88% in RES group	ISI	**Secondary outcome** - UC group: the median [Q1–Q3] scores were 9.5 [5.5; 20.8] at baseline and increased at 11.5 [3.8; 16.5] after intervention, with mean change (95% CI) from T0 to T1 at 0.5 (–6.3; 3.5) - HIIT Group: the median scores were 5.5 [3.0; 9.3] at baseline and increased at 9.0 [6.8; 10.8] after intervention, with mean change from T0 to T1 at 3.0 (1.5; 4.0) - RES group: the median scores were 8.5 [3.0; 12.8] at baseline and increased at 9.5 [6.0; 12.8] after intervention, with mean change from T0 to T1 at 1.0 (−2.0; 4.0) *NS difference between 3 groups after exercise program for insomnia*
Sheehan et al. 2020 [30]	To determine the effects of a 10-week EX intervention compared with a health education intervention on fatigue, quality of life outcomes and functional fitness in cancer survivors with documented fatigue. * To experience ongoing fatigue (score < 45 points) on the FACT-F	Exercise group Health education group	Type: [Supervised and home-based] progressive aerobic training and stretching with majority of brisk walking for fatigued patients (RPE at 11–13, 66–85 HR max) Frequency: twice weekly Intensity: None Time: 60 min Duration: 10 weeks Group-based fatigue management sessions, emphasizing non-exercise strategies Time: 60 min Frequency: once weekly Duration: 10 weeks	Baseline, post intervention (week 10), follow-up at 16 weeks post-intervention (26 weeks)	Adherence rate: 100% (10 weeks)	ISI	**Secondary outcome** - EX group: ISI scores decreased at each time: 15.2 ± 1.9 at baseline to 8.2 ± 1.7 in post intervention to 6.4 ± 1.6 at follow-up *Pre* vs. *post intervention*, *p = 0.001 and pre* vs. *26 weeks follow-up*, *p < 0.001* - Health education group: ISI scores decreased: 15.5 ± 1.9 at baseline to 13.3 ± 1.7 in post intervention *No time effect*
Yamada et al. 2021 [31]	To compare the effect of exercise (12 weeks) on psychosocial health in paired versus individually trained cancer patients.	Exercise in pairs and individually	Type: [Supervised], cardiovascular training, resistance training exercises (5–7 exercises), balance and flexibility Frequency: 3 times/week Intensity: 40–60% of HR reserve and 40–60% of 1-RM, RPE at 3-6 Time: 90 min Duration: 12 weeks	pre- (baseline), mid- (6-weeks), and post (12 weeks) intervention	Adherence rate: 78.5% for individually trained patients and 85.7% for paired patients	ISI	**Secondary outcome** - Individually group: ISI scores: 11 ± 8.3 at pre intervention to 10.4 ± 7.8 at mid intervention to 10.3 ± 9 in post intervention *No time effect* - Paired group: ISI scores: 9.8 ± 6.9 at pre intervention to 7 ± 6.3 at mid intervention to 7.3 ± 6.3 in post intervention *Pre* vs. *mid intervention, p < 0.05*

* indicates that for some studies there were specific inclusion criteria regarding sleep and fatigue. aSAH: aneurysmal subarachnoid haemorrhage; Avg: average; CBT-I: cognitive behavioral therapy for insomnia; CI: confidence interval; CTRF: cancer-treatment related fatigue; CX: chemotherapy; ES: effect size; EX: exercise; FACT-F: Functional Assessment of Cancer Therapy-Fatigue; HIIT: high intensity interval training; HR: heart rate; ISI: Insomnia Severity Index; NACRT: neoadjuvant chemoradiotherapy; PA: physical activity; Q1–Q3: quartile 1–quartile 3; QoL: quality of life; RES: resistance; Rev/min: revolution/minute; RM: repetition maximum; RPE: Rating of Perceived Exertion; RX: radiotherapy; SE: sleep efficiency; SOL: Sleep Onset Latency; THRmax: theoretical maximal heart rate; TWT: total wake time; UC: usual care; WASO: wakefulness after sleep onset.

## Data Availability

The data presented in this study are available in this article and Supplementary Materials.

## Supplementary Materials

The following supporting information can be downloaded at: https://www.mdpi.com/article/10.3390/cancers16122241/s1, Figure S1: Search strategy PubMed; Figure S2: Search strategy Cochrane Library.
