# Supplementary material for: Exercise Intervention on Insomnia in Patients with a Cancer: A Systematic Review of the Literature
Source: Cancers (Basel). 2024 Jun 17;16(12):2241. doi: 10.3390/cancers16122241 (PMC11201959; doi:10.3390/cancers16122241)
Supplement: Supplementary file 1 [file cancers-16-02241-s001.zip › cancers-3014731-supplementary.pdf]

Exercise Intervention on Insomnia in Patients with a Cancer: A Systematic Review of the Literature

Chloé Drozd [1,2,3,*], Elsa Curtit [4,5], Valérie Gillet [3], Quentin Jacquinot [2,6], Nathalie Meneveau [5] and Fabienne Mougin [1,2]

1 Sports Science Faculty, University of Franche-Comté, 25000 Besançon, France

2 Research Unit SINERGIES, University of Franche-Comté, 25000 Besançon, France

3 Sleep Medicine Center, Don Du Souffle Association, 25000 Besançon, France

4 INSERM U1098 Right, University of Franche-Comté, 25000 Besançon, France

5 Department of Medical Oncology, University Hospital, 25000 Besançon, France

6 Regional Federative Cancer Institute of Franche-Comté, 25000 Besançon, France

* Correspondence: chloe.drozd@univ-fcomte.fr

1 (((neoplasms[MeSH Terms]) OR (neoplasm*[Title/Abstract])) OR (cancer*[Title/Abstract])) OR (malignan*[Title/Abstract])

2 (Sleep Initiation and Maintenance Disorders[MeSH Terms]) OR (insomnia*[Title/Abstract])

3 (((((((((((((((Exercise[MeSH Terms]) OR (gymnastics[MeSH Terms])) OR (Physical Conditioning, Human[MeSH Terms])) OR (Endurance Training[MeSH Terms])) OR (Resistance Training[MeSH Terms])) OR (Running[MeSH Terms])) OR (Jogging[MeSH Terms])) OR (Sports[MeSH Terms])) OR (exercise therapy[MeSH Terms])) OR (Swimming[MeSH Terms])) OR (Walking[MeSH Terms])) OR (Nordic Walking[MeSH Terms])) OR (Dancing[MeSH Terms])) OR (Dance Therapy[MeSH Terms])) OR (Cycling[MeSH Terms])) OR (Exercise Movement Techniques[MeSH Terms])) OR (Rehabilitation[MeSH Terms])) OR (physical education and training[MeSH Terms])

4 ((((((((((((((Exercise[Title/Abstract]) OR ("Physical Activit*"[Title/Abstract])) OR (Aerobic[Title/Abstract])) OR (Gymnast*[Title/Abstract])) OR (training[Title/Abstract])) OR (Running[Title/Abstract])) OR (Jogging[Title/Abstract])) OR (Sport*[Title/Abstract])) OR (Swimming[Title/Abstract])) OR (Walking[Title/Abstract])) OR (Dancing[Title/Abstract])) OR (Dance[Title/Abstract])) OR (Pilates[Title/Abstract])) OR (bicycling[Title/Abstract])) OR ("physical education"[Title/Abstract])) OR (rehabilitation[Title/Abstract])

5 #3 OR #4

6 #1 AND #2 AND #5

Figure S1. Search strategy PubMed.

#1	MeSH descriptor: [Neoplasms] explode all trees
#2	neoplasm* OR cancer* OR malignan*
#3	#1 OR #2
#4	MeSH descriptor: [Sleep Initiation and Maintenance Disorders] explode all trees
#5	insomnia*
#6	#4 OR #5
#7	MeSH descriptor: [Exercise] explode all trees
#8	MeSH descriptor: [Gymnastics] explode all trees
#9	Physical Conditioning, Human
#10	Endurance Training
#11	MeSH descriptor: [Resistance Training] explode all trees
#12	MeSH descriptor: [Running] explode all trees
#13	MeSH descriptor: [Jogging] explode all trees
#14	MeSH descriptor: [Sports] explode all trees
#15	MeSH descriptor: [Exercise Therapy] explode all trees
#16	MeSH descriptor: [Swimming] explode all trees
#17	MeSH descriptor: [Walking] explode all trees
#18	MeSH descriptor: [Dancing] explode all trees
#19	MeSH descriptor: [Dance Therapy] explode all trees
#20	MeSH descriptor: [Exercise Movement Techniques] explode all trees
#21	MeSH descriptor: [Rehabilitation] explode all trees
#22	MeSH descriptor: [Physical Education and Training] explode all trees
#23	Exercise OR "Physical Activit*" OR "Aerobic" OR Gymnast* OR training OR Running OR Jogging OR Sport* OR Swimming OR Walking OR Dancing OR Dance OR pilates Or Rehabilitation OR "physical education"
#24	#7 OR #8 OR #9 OR #10 OR #11 OR #12 OR #13 OR #14 OR #15 OR #16 OR #17 OR #18 OR #19 OR #20 OR #21 OR #22 OR #23
#25	#3 AND #6 AND #24

Figure S2. Search strategy Cochrane Library.
